# Audit of Psychiatric History Taking and Mental State Examination Clerking by Psychiatric Trainee Doctors in North Wales

**DOI:** 10.1192/bjo.2026.11295

**Published:** 2026-06-30

**Authors:** Saima Mustafa, Rajvinder S Sambhi, Anna Walsh, Saqib Rashiq, Georgia Fotiou

**Affiliations:** 1 Betsi Cadwaladr University Health Board, Bodelwyddan, United Kingdom; 2 Betsi Cadwaladr University Health Board, Wrexham, United Kingdom; 3 Betsi Cadwaladr University Health Board, Gwynedd, United Kingdom

## Abstract

**Aims:**

To improve the quality of psychiatric history taking and mental state examination by psychiatric trainee doctors in North Wales.

**Methods:**

A retrospective psychiatric case note audit was conducted across three acute psychiatric inpatient units in North Wales (Wrexham, Rhyl and Bangor sites). A total of 35 inpatient records were reviewed (Wrexham n=15, Rhyl n=10, Bangor n=10) against standards derived from the *Oxford Textbook of Psychiatry* chapter on history taking and mental state examination. Quality was scored (0–2) against 60 essential criteria.

**Results:**

The aggregate compliance to the standards was 50.6%, demonstrating significantly less than the 100% target for full documentation. While administrative tasks (84.3%) and objective MSE findings (68.5%) were relatively well-documented, complex narrative elements such as HOPC especially core symptoms of anxiety (10.0%), depression (26.8%),and past psychiatric history (22.9%) showed critical gaps. The History of Presenting Complaint achieved only 26.4% compliance. Most significantly, while risk assessment was documented in 58.1% of cases, the translation into an actionable risk management plan was documented in only 8.6% patients.

**Conclusion:**

There was variation in quality standards achieved at all the three acute psychiatric sites in North Wales. The resident trainee doctors used different admission clerking proformas at the three sites. The details captured in the admission clerking for new psychiatric admissions did not fully align with the details expected in a psychiatric history taking scenario as per the standards in the *Oxford* *Textbook* *of Psychiatry*. The missing information in history taking can contribute to suboptimal patient care including suboptimal risk management and such missing information and detail in history taking is seldom fully rectified later in the admission. We argue that the solution is not merely the introduction of another “tick-box” proforma, but the implementation of a standard with clear expectations to follow traditional detailed psychiatric history taking and mental state examination with clinical reasoning and synthesis, regardless of tool used for completing the clerking. Training sessions are being planned to be delivered to the trainee doctors with the aim of achieving 100% compliance in the re-audit ensuring that the “full story” is captured for every patient, every time.

